# Synthesis of a Cholesterol Derivative and Its Application in Gel Emulsion Preparation

**DOI:** 10.3390/molecules29246055

**Published:** 2024-12-23

**Authors:** Yang Liu, Shuaihua Liu, Qiang Zhang, Guanghui Tian

**Affiliations:** 1 School of Chemistry and Environment Science, Shaanxi University of Technology, Hanzhong 723001, China; 2 Shaanxi Key Laboratory of Catalysis, Shaanxi University of Technology, Hanzhong 723001, China

**Keywords:** small-molecule gelator, stabilizer, gel emulsion

## Abstract

As a small-molecule gelator used as a stabilizer in gel emulsions, it has numerous advantages, such as low dosage, independence from phase ratios, and ease of control. In this study, a cholesterol derivative (CSA) was designed and synthesized to be used as a stabilizer for gel emulsions. Gelation experiments demonstrated that this small molecule could gelate various organic solvents, including linear alkanes, toluene, isoamyl alcohol, and acetone. Based on these gelation experiments, a series of gel emulsions were prepared with water as the dispersed phase and an organic solvent immiscible with water as the continuous phase. Finally, the gelation behavior of the gelator/water/toluene and gelator/water/cyclohexane systems was investigated, exploring the effects of different systems and varying water content within the same system on the structure and stability of the gel emulsions. Studies have shown that the gel emulsion prepared from the gelator/water/toluene system exhibits superior stability, likely due to the molecular self-assembly behavior of this cholesterol derivative exhibited in the water/toluene biphasic system. The research results provide a basis for using gel emulsions as templates to prepare porous materials and adjust their internal structure, ultimately laying a solid foundation for applying these porous materials in fields such as adsorption and catalysis.

## 1. Introduction

Gel emulsions, also known as high internal phase emulsions, are typically composed of a stabilizer, a dispersed phase, and a continuous phase. A gel network structure forms between the dispersed and continuous phases, giving the emulsion a gel-like state. Currently, gel emulsions are mainly classified according to two criteria: (1) Based on the polarity differences between the dispersed and continuous phases, they can be divided into water-in-oil (W/O), oil-in-water (O/W), and multiple emulsion (W/O/W or O/W/O) types of gel emulsions. (2) Based on the type of stabilizer used, they can be categorized into gel emulsions stabilized by solid micro/nano particles (also known as Pickering emulsions), surfactants, or small-molecule gelators [1,2,3]. Compared to traditional gel emulsions, gel emulsions stabilized by small-molecule gelators do not require an internal phase volume fraction greater than 74% or the addition of large amounts of small-molecule gelators to achieve stability. The stabilization mechanism of gel emulsions with small-molecule gelators involves self-assembly through weak intermolecular interactions (such as hydrogen bonding, π-π stacking, van der Waals forces, electrostatic interactions, and host–guest interactions) to form a three-dimensional network structure, restricting solvent mobility and resulting in a stable gel emulsion [4,5,6]. By introducing specific functional groups into the structure of small-molecule gelators, gel emulsions can become responsive to stimuli such as light, electricity, and pH. This makes them highly valuable for applications in drug delivery and release, tissue engineering, catalysis, food, and other fields [7,8,9,10].

It is well known that cholesterol derivatives, like cholesterol itself, have characteristics such as a rigid structure, multiple chiral centers, and strong intermolecular interactions. Therefore, they are commonly important structural units in small-molecule gelators. Since the 1980s, they have received widespread attention and continue to be of great interest to scientists today [11,12]. For example, in previous studies on gel emulsions, many research groups used cholesterol derivatives as stabilizers to effectively gelate oil–water mixtures at room temperature [13,14,15,16]. Unlike traditional gel emulsions, gel emulsions stabilized by small-molecule gelators have a continuous phase that is gel-like, with the dispersed phase physically encapsulated within the gel network. However, not all organogels can serve as the continuous phase; stable gel emulsions can only form if the following three conditions are met: (1) the gelator must be insoluble in water; (2) the organic phase must be immiscible with water; (3) the interfacial energy must be low. Compared to the other two types of stabilizers (surfactants and solid micro/nano particles), small-molecule gelators offer the following advantages: (1) low usage; (2) no need for the continuous phase volume to exceed 74%; (3) availability of reactive groups on the surface, among others [17,18,19].

**Figure 1:**
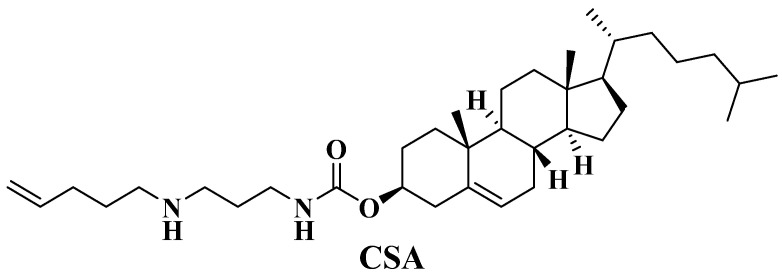
Structure of CSA.

Based on cholesterol derivatives (CSA, Figure 1), this experiment produced a series of polymerizable gel emulsions, investigating the effects of different solvent systems and water content on gel emulsion stability and characterizing the internal structure of the gel emulsions. The gel emulsions stabilized by cholesterol derivatives offer advantages such as low stabilizer usage and high stability.

## 2. Results and Discussion

### 2.1. Gelation Behaviors

The gelation behavior of CSA was tested in 28 solvents at a standard concentration of 3% (*w*/*v*), and the results are shown in Table 1. The results indicate that CSA did not gelate any of the tested solvents at room temperature (20 °C); however, under ultrasonic conditions, it was able to gel conventional solvents such as toluene, *n*-hexane, and *n*-heptane, which are alkanes, and it was immiscible with water. It is well known that small-molecule gelators can gel certain organic solvents while being immiscible with water. Therefore, this gelators/water/organic solvents system may have the potential to form gel emulsions.

**Figure 2:**
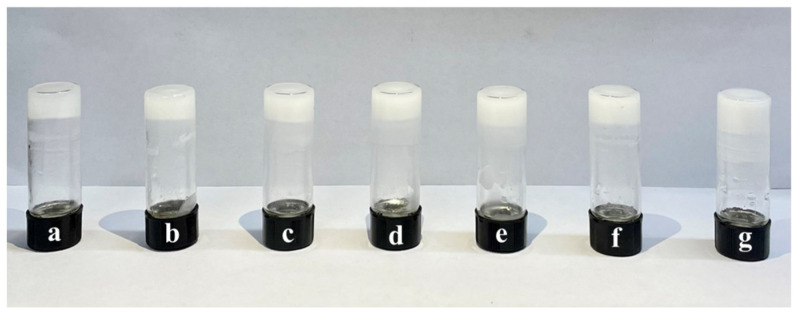
Gel emulsion images formed in different solvents: (**a**) *n*-hexane, (**b**) *n*-heptane, (**c**) *n*-octane, (**d**) *n*-nonane, (**e**) *n*-decane, (**f**) cyclohexane, and (**g**) toluene (basic composition: CSA at 2.5% of the organic phase by weight, 100 µL organic phase, 900 µL water).

Based on the above gelation experiment results, the stabilizer CSA (at a concentration of 2.5%, *w*/*v* for the continuous phase) was added to 100 µL of a gelable organic solvent, followed by the addition of 900 µL of ultrapure water. The mixture was shaken to ensure thorough mixing of the oil and water phases, and after standing at room temperature (20 °C), a gel emulsion was obtained. The experimental results are shown in Figure 2. It is noteworthy that the gelator forms a semi-gel in cyclohexane, but after adding ultrapure water in a volume ratio of 1:9 (cyclohexane/water) and stirring for several minutes, a stable, non-flowing gel emulsion can also be formed.

As can be seen above, this gel emulsion preparation method is simple, and the amount of stabilizer used is low, only 2.5% (*w*/*v*) of the oil phase volume. Moreover, it exhibits good storage stability, remaining stable for several weeks to months at room temperature (20 °C). These advantages lay a solid foundation for practical applications.

### 2.2. The Effect of Water Content on Gel Emulsion

**Figure 3:**
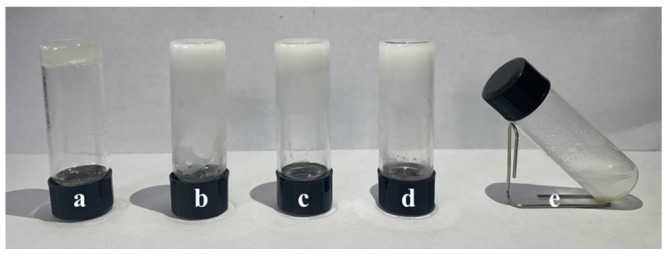
Phase behavior of CSA/water/toluene (total volume: 1 mL) with different water contents: (**a**) 0%, (**b**) 30%, (**c**) 60%, (**d**) 90%, and (**e**) 100%.

**Figure 4:**
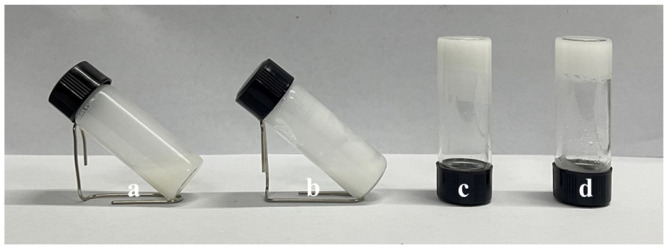
Phase behavior of CSA/water/cyclohexane (total volume: 1 mL) with different water contents: (**a**) 30%, (**b**) 60%, (**c**) 80%, and (**d**) 90%.

The internal structure of the gel emulsion is closely related to its water content, which also affects the stability of the gel emulsion. Therefore, we investigated the effect of water content on the phase behavior of the gel emulsion system. Based on the results of the gelation experiments, the CSA/water/toluene system and the CSA/water/cyclohexane system were selected for study, as shown in Figure 3 and Figure 4.

As shown in Figure 3, for the CSA/water/toluene system, the gelator is able to gel toluene. Additionally, the gelator is almost insoluble in water, and the volume fraction of the water phase in the dispersion can be adjusted over a wide range. Similarly, for the CSA/water/cyclohexane system, we also investigated the effect of water content on the phase behavior of the gel emulsion. Previous gelation experiments showed that under ultrasonic conditions at room temperature (20 °C), the system ultimately formed only a semi-gel, which remained flowable. When preparing the gel emulsion, if the water content in the system was below 74%, the system was a uniform, flowable white emulsion. However, when the water content was 80% or 90%, the system lost its flowability and formed a gel emulsion, as shown in Figure 4. By comparing these two systems, it can be seen that the CSA/water/toluene system is not restricted by the traditional gel emulsion requirement that the volume fraction of the dispersed phase must be greater than 74%. The dispersed phase volume fraction can be adjusted over a wide range, allowing stable gel emulsion systems to form from 30% to 90%, thus enabling a broad range of structural and property adjustments for the practical application of gel emulsions. On the other hand, the CSA/water/cyclohexane system exhibits the characteristics of a traditional gel emulsion system, where the volume fraction of the dispersed phase must be greater than 74% to form a gel emulsion. This traditional gel emulsion relies on the close packing of droplets to make the system lose its flowability [20].

**Figure 5:**
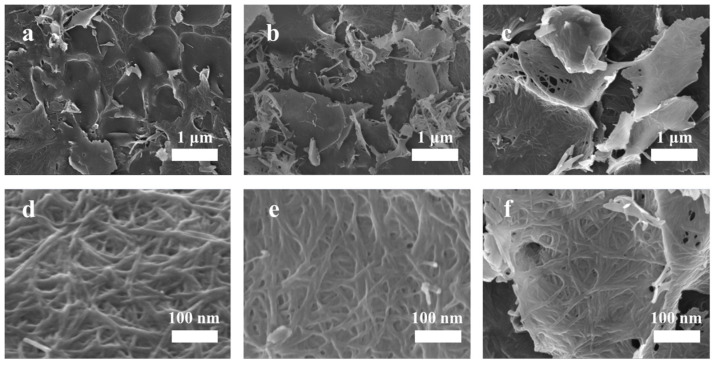
SEM images of the xerogels prepared from different gel emulsions of CSA/water/toluene (total volume, 1 mL) of different water contents: (**a**) 30%, (**b**) 60%, and (**c**) 90%, and its locally enlarged view (**d**), (**e**), (**f**), respectively.

**Figure 6:**
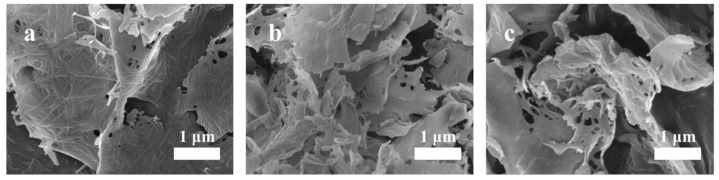
(**a**) SEM image of the xerogel from the CSA/water/toluene system with 90% water content; (**b**,**c**) are SEM images of the xerogels prepared from CSA/water/cyclohexane gel emulsions with water contents of 80% and 90%, respectively (total volume: 1 mL).

To analyze the effect of water content on the structure of gel emulsions in the two different systems, the gel emulsions of the CSA/water/toluene and CSA/water/cyclohexane systems with varying water contents were selected, and SEM tests were conducted on the freeze-dried xerogels. As shown in Figure 5 and Figure 6, the microstructure of the xerogels from the gel emulsions changes with water content. For the CSA/water/toluene system, as the water content increases, the traces of water droplet pores in the aggregated structure become more pronounced (Figure 5a–c). It should be noted that SEM images show that the xerogels of this system contain layered structures with a fine fibrous mesh formed by the self-assembly of the small-molecule gelator. This structure may contribute to the formation of a stable gel emulsion, allowing it to remain stable even with a wide range of water content variations. As water content increases, the mesh structure in the layers of this system becomes less prevalent (Figure 5d–f). This phenomenon may be due to the reduced volume of the oil phase as water content increases, which lowers the gelator concentration in the overall gel emulsion system, resulting in greater porosity and fewer network structures. For the CSA/water/cyclohexane system, only xerogels with 80% and 90% water content were tested, as gel emulsions cannot form when the water content is below 74%. From the SEM images in Figure 6, it can be seen that the morphology of the CSA/water/cyclohexane system primarily consists of stacked sheet-like structures. Unlike the CSA/water/toluene system, which contains a fibrous mesh structure within the layers, this structure is absent in the CSA/water/cyclohexane system. The loss of the fibrous mesh structure’s stabilizing effect on the gel emulsion may explain why the dispersed phase in the cyclohexane system needs to exceed 74% to form a gel emulsion.

### 2.3. Rheological Studies

Gel emulsions, as a type of soft material, are commonly characterized for their stability through rheological measurements. In this study, the stabilizer content was kept constant at 2.5% (*w*/*v*) relative to the continuous phase, and the storage modulus (G’) and loss modulus (G’’) of CSA/toluene/water and CSA/cyclohexane/water systems with varying water contents were tested as functions of shear stress. For the CSA/toluene/water gel emulsion system with different water contents (Figure S8a), G’ was higher than G’’ in the low shear stress range, indicating a gel-like elastic behavior resembling that of a solid. When the shear stress exceeded the yield stress, both G’ and G’’ dropped sharply, with G’’ becoming higher than G’, signifying a transition to a fluid-like viscous behavior and indicating the destruction of the gel emulsion structure. It was also observed that as the water content (*v*/*v*) decreased (90%, 60%, 30%), both G’ and G’’ increased, with values of approximately ~98 Pa, ~2414 Pa, and ~5060 Pa for G’, and ~9 Pa, ~388 Pa, and ~948 Pa for G’’, respectively. The yield stress also increased, with values of 12 Pa, 25 Pa, and 27 Pa. These results indicate that water content significantly affects the stability and viscoelasticity of the gel emulsion system. Based on these observations, it is hypothesized that the continuous phase of this system may consist of a three-dimensional gel network structure, within which the dispersed phase is physically encapsulated. This structural characteristic suggests that the mechanical strength of the gel emulsion is dependent on the strength of the continuous phase. The more continuous phase present, the thicker the walls encapsulating the dispersed phase, leading to higher G’ and yield stress. This behavior contrasts with the conventional gel emulsion systems, where larger dispersed phase volumes typically result in greater stability [21,22]. This hypothesis is further supported by the SEM observations of the continuous phase’s three-dimensional network structure (Figure 5). In comparison, the CSA/cyclohexane/water system exhibited similar characteristics to the CSA/toluene/water system (Figure S8b). As the water content (*v*/*v*) decreased from 90% to 80%, both G’ and G’’ increased, with G’ values of approximately ~168 Pa and ~742 Pa, and G’’ values of ~10 Pa and ~83 Pa, respectively. Additionally, the results clearly show that the G’ and G’’ values of the CSA/toluene/water system were generally higher than those of the CSA/cyclohexane/water system, indicating that the stability of the CSA/toluene/water system is overall superior. This finding is consistent with the results of the inversion experiments conducted earlier (Figure 3 and Figure 4).

## 3. Experimental Section

### 3.1. Reagents and Instruments

1,3-Propanediamine (≥98%), cholesterol chloroformate (≥98%), and 5-bromo-1-pentene (≥98%) were purchased from Shanghai Meryer Biochemical Technology Co., Ltd. (Shanghai, China) and used without further purification. Triethylamine (≥99.5%) was obtained from Saen Chemical Technology (Shanghai) Co., Ltd. (Shanghai, China). Tetrahydrofuran was distilled after refluxing over sodium, dichloromethane (DCM) was distilled over CaH_2_, and *n*-hexane, methanol, and ethyl acetate were purified by distillation before use. The laboratory water was ultrapure water purified through a UP ultrapure water system.

SCR1-type thermostatic magnetic stirrer (BIOCOTE Ltd., Coventry, UK); C20-type electric thermostatic drying oven (Shanghai Ledon Industrial Co., Ltd., Shanghai, China); Xinyi-10N freeze dryer (Ningbo Xinyi Ultrasonic Equipment Co., Ltd., Ningbo, China); ADVANCE 600 fourier digital NMR spectrometer (BRUKER, Fällanden, Switzerland); JSM-7610F scanning electron microscope (JEOL, Tokyo, Japan); VERTEX70 infrared spectrometer (BRUKER, Fällanden, Switzerland); high-resolution mass spectra (Thermo Fisher Scientific, Waltham, MA, USA); ARES-G2 rheometer (TA Instruments, New Castle, DE, USA).

### 3.2. Experimental Procedure

#### 3.2.1. Synthesis and Characterization of the Gelator CSA

**Scheme 1:**
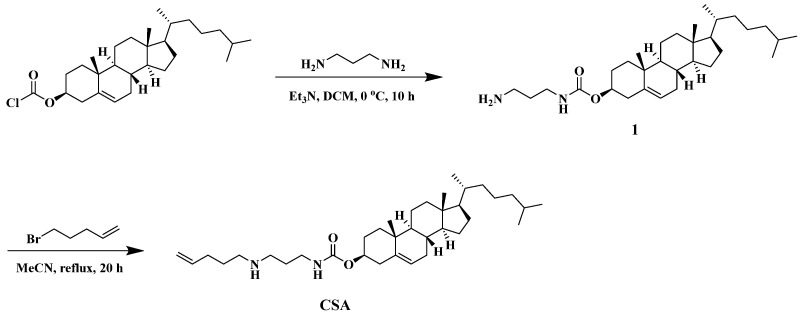
Synthesis route of the CSA.

The synthesis process of the gelator CSA is shown in Scheme 1.

##### Synthesis of Intermediate 1

To a 100 mL of purified dichloromethane, add 2.5 mL (30 mmol) of 1,3-propanediamine and 0.21 mL (1.5 mmol) of triethylamine, then stir the mixture at 0 °C under an argon atmosphere. Dissolve 0.68 g (1.5 mmol) of cholesterol chloroformate in 30 mL of dichloromethane, and slowly add this solution to the above mixture of 1,3-propanediamine and triethylamine using a constant-pressure dropping funnel. After the addition is complete, stir the mixture at room temperature (20 °C) for 10 h, filter, and wash the filtrate five times with saturated saline. Then, dry the filtrate over anhydrous sodium sulfate, remove the dichloromethane by rotary evaporation, and dry the residue under vacuum to obtain a white solid, which is intermediate 1 with a yield of 93%. Intermediate 1 can be used directly in the following synthesis without further purification [4].

##### Synthesis of CSA

Dissolve 0.74 g (1.5 mmol) of intermediate 1 in 40 mL of acetonitrile, then slowly add 90 µL (0.75 mmol) of 5-bromo-1-pentene. Reflux the mixture at 80 °C for 20 h, then cool it to room temperature (20 °C). Add an appropriate amount of dichloromethane, wash the mixture three times with saturated saline, dry over anhydrous sodium sulfate, and remove the solvent by rotary evaporation. Perform column chromatography (silica gel, dichloromethane/methanol = 15/1) to obtain the CSA as a white powder with a yield of 60% [23].

##### Characterization of Intermediate 1 and CSA

Intermediate 1. ^1^H NMR (600 MHz, CDCl_3_) δ 5.37 (s, 1H, alkenyl), 5.16 (t, 1H, N*H*CO), 4.48 (m, 1H, oxycyclohexyl), 3.27–3.21 (m, 2H, NH*CH*_2_), 2.78 (t, 2H, NH_2_*CH*_2_), 2.33–0.67 (m, 47H, cholesteryl protons, N*H*_2_ and C*H*_2_). ^13^C NMR (150 MHz, CDCl_3_) δ 156.36, 139.84, 122.44, 74.20, 56.67, 56.13, 49.99, 42.29, 39.73, 39.66, 39.51, 38.81, 38.59, 37.00, 36.55, 36.17, 35.79, 33.08, 31.89, 31.86, 28.23, 28.18, 28.00, 24.28, 23.84, 22.85, 22.54, 21.04, 19.34, 18.71, 11.86. HRMS (ESI-TOF): *m*/*z* [M + H]^+^ calcd. for C_31_H_55_N_2_O_2_^+^ 487.42581, found 487.42560. IR: 3350 cm^−1^ (N-H), 1700 cm^−1^ (C=O), 1600 cm^−1^ (C=C). M.p.: 164.8 °C.

CSA. ^1^H NMR (600 MHz, CDCl_3_) δ 5.76 (ddt, 1H, CH_2_=C*H*(CH_2_)_3_), 5.37–5.36 (m, 2H, alkenyl and N*H*CO), 5.09 (d, 1H, C*H*_2_=CH), 5.04 (d, 1H, C*H*_2_=CH), 4.50–4.41 (m, 1H, oxycyclohexyl), 3.35 (t, 2H, C*H*_2_NHCO), 3.02 (t, 2H, C*H*_2_NHCH_2_), 2.94 (t, 2H, CH_2_NHC*H*_2_), 2.30–0.67 (m, 50H, cholesteryl protons and CH_2_N*H*CH_2_). ^13^C NMR (150 MHz, CDCl_3_) δ 157.5, 139.8, 136.3, 122.8, 116.6, 75.1, 56.8, 56.2, 50.1, 47.7, 45.2, 42.4, 39.8, 39.6, 38.6, 37.3, 37.0, 36.6, 36.3, 35.9, 32.0, 31.9, 30.7, 28.3, 28.2, 28.1, 26.6, 25.0, 24.4, 23.9, 22.9, 22.7, 21.1, 19.4, 18.8, 12.0. HRMS (ESI-TOF): *m*/*z* [M + H]^+^ calcd. for C_36_H_63_N_2_O_2_^+^ 555.48841, found 555.48775. IR: 3200 cm^−1^ (N-H), 1680 cm^−1^ (C=O), 1600 cm^−1^ (C=C). M.p.: 251.6 °C.

#### 3.2.2. Gelation Experiment

Take 0.015 g of CSA and 0.5 mL of the test gel solvent, and place them in a sealed sample tube (1 cm in diameter, 3 cm in height). After ultrasonic treatment at room temperature (20 °C) for 20 min, allow the mixture to stand and invert the sample bottle to observe whether a gel has formed. The gel obtained by ultrasonication at room temperature is denoted as G. If no gel forms, heat the sample bottle until the solid is completely dissolved, then cool it to room temperature and invert the sample bottle to observe its state. The results are recorded as follows: G (gel) for gel formation; S (solution) for a solution; P (precipitate) for systems that dissolve upon heating but precipitate upon cooling; PG (partially gelled) for mixtures of gel and solution; and I (insoluble) for systems that cannot dissolve at the boiling point of the solvent (Table 1).

#### 3.2.3. Preparation of Gel Emulsion

Add the stabilizer CSA (at a concentration of 2.5% *w*/*v* for the continuous phase) to the oil phase. After the gelator completely dissolves at room temperature (20 °C) or under mild heating, add the appropriate amount of ultrapure water. Then, place the mixture on a homogenizer set to a constant speed of 2500 rpm and shake vigorously to form a viscous solution, ensuring thorough mixing of the oil and water phases. After standing for about 3–5 min, invert the test tube to observe the loss of fluidity, resulting in the formation of a W/O-type gel emulsion. The compositions of the gel emulsions are listed in Table 2.

#### 3.2.4. *T*_gel_ Measurements

After preparing the gel in a small pressure-resistant tube, a small steel ball weighing less than 100 mg is placed on top of the gel using magnetic manipulation. The gel is then heated in an oil bath at a heating rate of 1 °C/min. When the gel transitions into a sol, the steel ball begins to move downward, and the gel’s phase transition temperature (*T*_gel_) is determined simultaneously [24].

#### 3.2.5. Rheological Measurements

The rheological properties of the gel emulsions were measured using a stress-controlled rheometer (TA Instruments, ARES-G2) equipped with geometrically constructed steel-coated parallel plate (20 mm in diameter). The gap between the two plates was set to 1.0 mm, and the temperature was maintained at 20 °C. A solvent trapping device was placed above the plate to minimize solvent evaporation during the measurement. Stress sweep at a constant frequency (1 Hz) was performed, which provided information about the linear viscoelastic region of the gel emulsions sample.

## 4. Conclusions

In summary, we synthesized a new cholesterol derivative (CSA) and conducted gelation experiments to prepare gel emulsions using two-phase systems of immiscible organic solvents and water. The gelation experiments showed that the small-molecule gelator prepared in this study could gel various organic solvents (such as linear alkanes, toluene, isopentanol, and acetone). Based on these gelation experiments, a series of stable gel emulsions were prepared using gelable organic solvents as the continuous phase and water as the dispersed phase. Finally, we investigated the gelation behavior of the CSA/water/toluene and CSA/water/cyclohexane systems, exploring the effects of different systems and different water contents within the same system on the structure and stability of gel emulsions. In future application studies, we plan to use this gel emulsion as a template to prepare porous materials. By adjusting the ratio of the dispersed phase to the continuous phase, we aim to control the internal structure of the porous material and test its performance in adsorption, catalysis, and related applications.

## Figures and Tables

**Table 1:** CSA behavior of gelator (3%, *w*/*v*) in different solvents, gel–sol phase transition temperature (*T*_gel_/°C).

Solvent	Result	Solvent	Result	Solvent	Result	
methanol	P	petroleum ether	I	*n*-hexane	G, ~63 ^a^	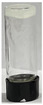
ethanol	P	DCM	S	*n*-heptane	G, ~92	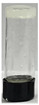
*n*-propanol	P	ethyl acetate	P	*n*-octane	G, ~113	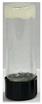
*n*-butanol	P	THF	S	*n*-nonane	G, ~72	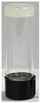
*n*-pentanol	P	pyridine	S	*n*-decane	G, ~86	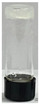
isopropanol	P	TEA	S	cyclohexane	PG	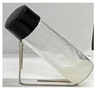
H_2_O	I	ethyl ether	PG	toluene	G, ~107	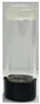
CCl_4_	S	DMSO	P	acetone	G	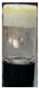
CHCl_3_	S	DMF	P	isopentanol	G	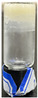
vinyl cyanide	P	acetic acid	P	acetonitrile	I	

S = Solution; P = Precipitation; I = Insoluble; G = Gel; PG = Partial Gel. ^a^ 63 means that the *T*_gel_ (gel–sol phase transition temperature) of the gel system is 63 °C.

**Table 2:** Compositions of the gel emulsions.

NO.	CSA (%, *w*/*v*)	Toluene (µL)	Cyclohexane (µL)	Water (µL)	Result
CSA_3-90_ ^a^	3	100	0	900	GE
CSA_3-60_	3	400	0	600	GE
CSA_3-30_	3	700	0	300	GE
CSA_3-90_	3	0	100	900	GE
CSA_3-80_	3	0	200	800	GE

^a^ CSA*_x-y_*, where *x* stands for the concentration of CEA in the organic phase and *y* the volume fraction of the dispersed phase; GE stands for gel emulsion.

## Data Availability

Data are contained within the article and Supplementary Materials.

## Supplementary Materials

The following supporting information can be downloaded at: https://www.mdpi.com/article/10.3390/molecules29246055/s1, Table S1: Gelation time of CSA in different organic solvents; Figure S1: ^1^H NMR spectrum of intermediate 1; Figure S2: ^13^C NMR spectrum of intermediate 1; Figure S3: ^1^H NMR spectrum of CSA; Figure S4: ^13^C NMR spectrum of CSA; Figure S5: ESI mass spectrum of intermediate 1; Figure S6: ESI mass spectrum of CSA; Figure S7: The FTIR spectra of intermediate 1 and CSA; Figure S8: (a) The variation curves of G’ and G’’ with shear stress for CSA/toluene/water gel emulsions with different water contents; (b) The variation curves of G’ and G’’ with shear stress for CSA/cyclohexane/water gel emulsions with different water contents. References [4,23] are cited in the Supplementary Materials.
