# Supplementary material for: Synthesis of a Cholesterol Derivative and Its Application in Gel Emulsion Preparation
Source: Molecules. 2024 Dec 23;29(24):6055. doi: 10.3390/molecules29246055 (PMC11679281; doi:10.3390/molecules29246055)
Supplement: Supplementary file 1 [file molecules-29-06055-s001.zip › molecules-3342035-supplementary.pdf]

# Supporting Information

*for*

## Synthesis of a Cholesterol Derivative and Its Application in Gel Emulsion Preparation

Yang Liu[1,2,*], Shuaihua Liu[1], Qiang Zhang[1,2], Guanghui Tian[1,2,*]

[1] School of Chemistry and Environment Science, Shaanxi University of Technology, Hanzhong 723001, China

[2] Shaanxi Key Laboratory of Catalysis, Shaanxi University of Technology, Hanzhong 723001, China

* Correspondence: liuyang@snut.edu.cn (Y.L.); tiangh@snut.edu.cn (G.T.)

**CONTENS**

**1. Experimental Section**...…………………………………………………1

**2. Supplementary Tables and Figures**…………………………………………2

**3. References**……………………………………………………………………6

# 1. Experimental Section

## 1.1. Reagents and materials

1,3-Propanediamine (≥98%), cholesterol chloroformate (≥98%), and 5-bromo-1-pentene (≥98%) were purchased from Shanghai Meryer Biochemical Technolo-gy Co., Ltd. and used without further purification. Triethylamine (≥99.5%) was obtained from Saen Chemical Technology (Shanghai) Co., Ltd. Tetrahydrofuran was distilled after refluxing over sodium, dichloromethane (DCM) was distilled over CaH2, and n-hexane, methanol, and ethyl acetate were purified by distillation before use. The laboratory water was ultrapure water purified through a UP ultrapure water system.

## 1.2. Synthetic routes for CSA

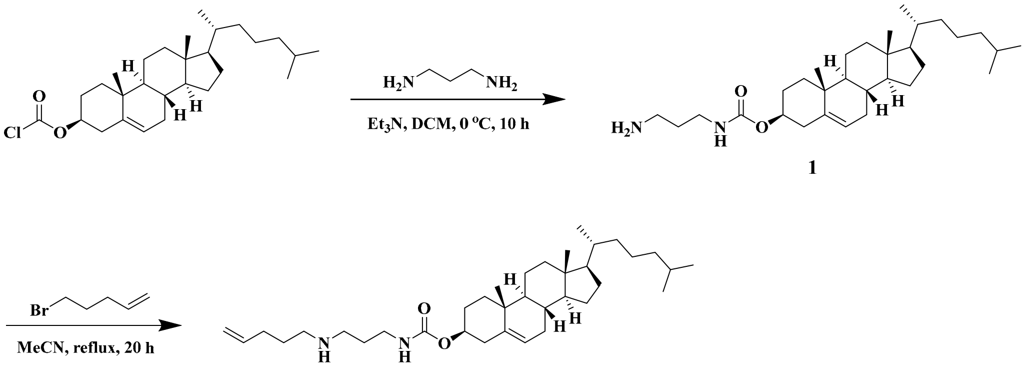


CSA

**Scheme S1.** The synthetic routes for CSA

## 1.3. Preparation of intermediate 1 and CSA

Intermediate 1 was prepared from the base-catalyzed condensation of 1,3-propanediamine with cholesterol chloroformate with referring a literature method.[1] CSA was prepared in similar ways as reported earlier.[2] The details are described below.

***Synthesis of intermediate 1:*** To a 100 mL of purified dichloromethane, add 2.5 mL (30 mmol) of 1,3-propanediamine and 0.21 mL (1.5 mmol) of triethylamine, then stir the mixture at 0°C under an argon atmosphere. Dissolve 0.68 g (1.5 mmol) of cholesterol chloroformate in 30 mL of dichloromethane, and slowly add this solution to the above mixture of 1,3-propanediamine and triethylamine using a constant-pressure dropping funnel. After the addition is complete, stir the mixture at room temperature for 10 h, filter, and wash the filtrate five times with saturated saline. Then, dry the filtrate over anhydrous sodium sulfate, remove the dichloromethane by rotary evaporation, and dry the residue under vacu-um to obtain a white solid, which is intermediate 1 with a yield of 93%. $^1$H NMR (600 MHz, $CDCl_3$) δ 5.37 (s, 1H, alkenyl), 5.16 (t, 1H, N*H*CO), 4.48 (m, 1H, oxycyclohexyl), 3.27-3.21 (m, 2H, NHC$H_2$), 2.78 (t, 2H, $NH_2$C$H_2$), 2.33-0.67 (m, 47H, cholesteryl protons, N$H_2$ and C$H_2$). $^{13}$C NMR (150 MHz, $CDCl_3$) δ 156.36, 139.84, 122.44, 74.20, 56.67, 56.13, 49.99, 42.29, 39.73, 39.66, 39.51, 38.81, 38.59, 37.00, 36.55, 36.17, 35.79, 33.08, 31.89, 31.86, 28.23,

28.18, 28.00, 24.28, 23.84, 22.85, 22.54, 21.04, 19.34, 18.71, 11.86. HRMS (ESI-TOF): *m/z* $[M + H]^+$ calcd. for $C_{31}H_{55}N_2O_2^+$ 487.42581, found 487.42560. IR: 3350 $cm^{-1}$ (N-H), 1700 $cm^{-1}$ (C=O), 1600 $cm^{-1}$ (C=C). M.p.: 164.8°C.

***Synthesis of CSA:*** Dissolve 0.74 g (1.5 mmol) of intermediate 1 in 40 mL of acetonitrile, then slowly add 90 μL (0.75 mmol) of 5-bromo-1-pentene. Reflux the mixture at 80°C for 20 h, then cool it to room temperature. Add an appropriate amount of dichloromethane, wash the mixture three times with saturated saline, dry over anhydrous sodium sulfate, and remove the sol-vent by rotary evaporation. Perform column chromatography (silica gel, dichloromethane/methanol = 15/1) to obtain the CSA as a white powder with a yield of 60%. $^1$H NMR (600 MHz, $CDCl_3$) δ 5.76 (ddt, 1H, $CH_2=CH(CH_2)_3$), 5.37-5.36 (m, 2H, alkenyl and N*H*CO), 5.09 (d, 1H, $CH_2$=CH), 5.04 (d, 1H, $CH_2$=CH), 4.50-4.41 (m, 1H, oxycyclohexyl), 3.35 (t, 2H, $CH_2$NHCO), 3.02 (t, 2H, $CH_2NHCH_2$), 2.94 (t, 2H, $CH_2NHCH_2$), 2.30–0.67 (m, 50H, cholesteryl protons and $CH_2NHCH_2$). $^{13}$C NMR (150 MHz, $CDCl_3$) δ 157.5, 139.8, 136.3, 122.8, 116.6, 75.1, 56.8, 56.2, 50.1, 47.7, 45.2, 42.4, 39.8, 39.6, 38.6, 37.3, 37.0, 36.6, 36.3, 35.9, 32.0, 31.9, 30.7, 28.3, 28.2, 28.1, 26.6, 25.0, 24.4, 23.9, 22.9, 22.7, 21.1, 19.4, 18.8, 12.0. HRMS (ESI-TOF): *m/z* $[M + H]^+$ calcd. for $C_{36}H_{63}N_2O_2^+$ 555.48841, found 555.48775. IR: 3200 $cm^{-1}$ (N-H), 1680 $cm^{-1}$ (C=O), 1600 $cm^{-1}$ (C=C). M.p.: 251.6°C.

### 1.4. Preparation of xerogels

A gel emulsion was transferred into liquid $N_2$, then frozen for 30 min, and then frozen at −40°C overnight. Finally, the frozen sample was freeze dried at vacuum (≤50 Pa) to yield dry xerogels.

### 1.5. SEM observation

Scanning electron microscopy (SEM) images of the xerogels were taken on a JSM-7610F environmental scanning electron microscope. The accelerating voltage was 5.0 kV and the emission current was 100 μA. The xerogels to be examined were cut into slice first, and were observed from the section.

## 2. Supplementary Tables and Figures

**Table S1** Gelation time of CSA in different organic solvents.

| Solvent | Time (Result) | Solvent | Time (Result) | Solvent | Time (Result) |
|---|---|---|---|---|---|
| toluene | 10 s (G) | *n*-hexane | 3 min (G) | *n*-nonane | 5 min (G) |
| acetone | 5 min (G) | *n*-heptane | 3 min (G) | *n*-decane | 5 min (G) |
| isopentanol | 5 min (G) | *n*-octane | 5 min (G) | cyclohexane | 20 min (PG) |

G = Gel; PG = Partial Gel.

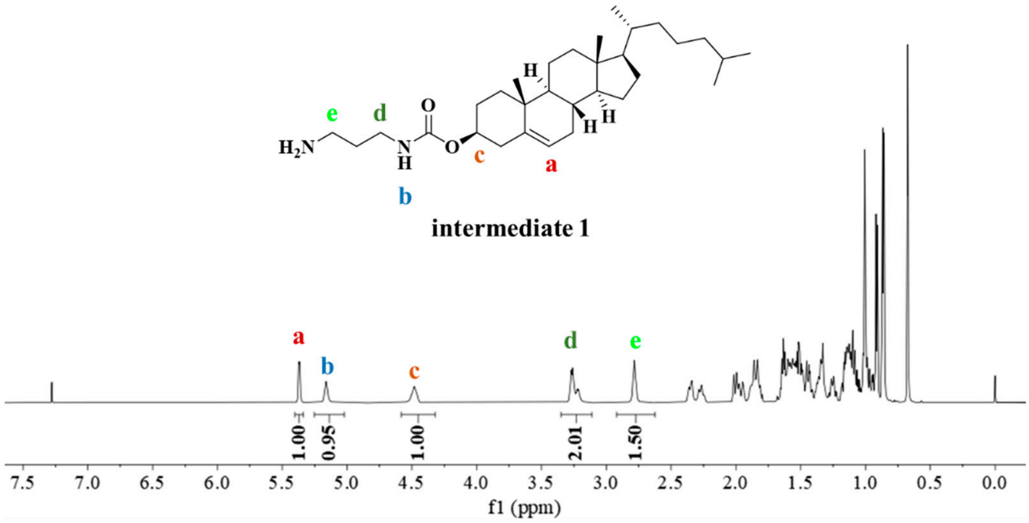


**Figure S1** $^{1}$H NMR spectrum of intermediate 1.

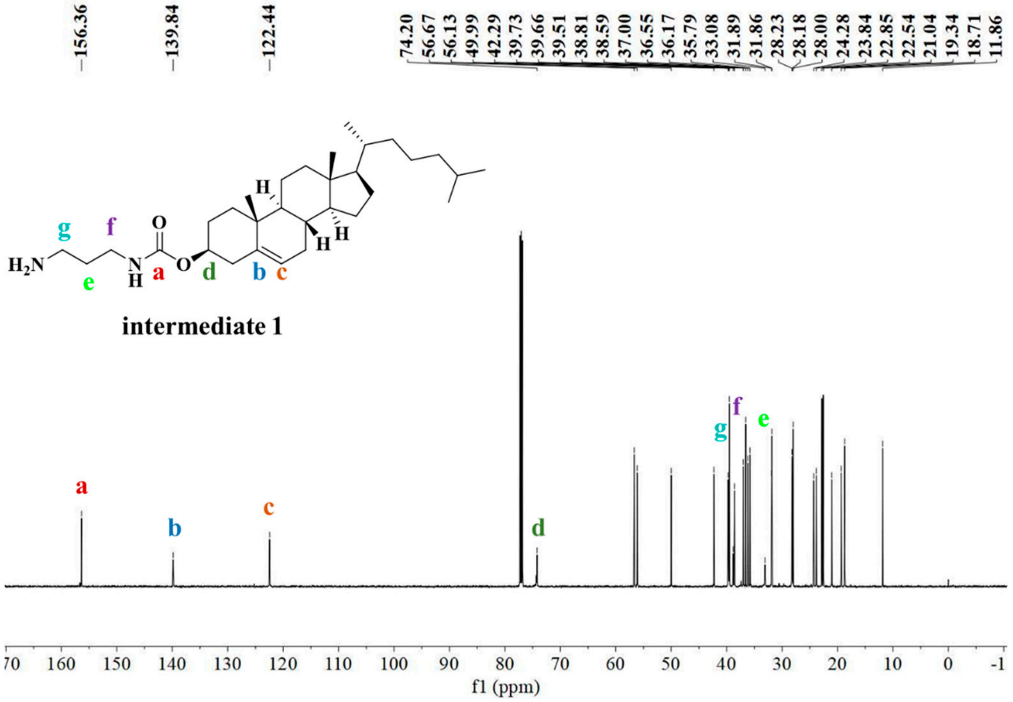


**Figure S2** $^{13}$C NMR spectrum of intermediate 1.

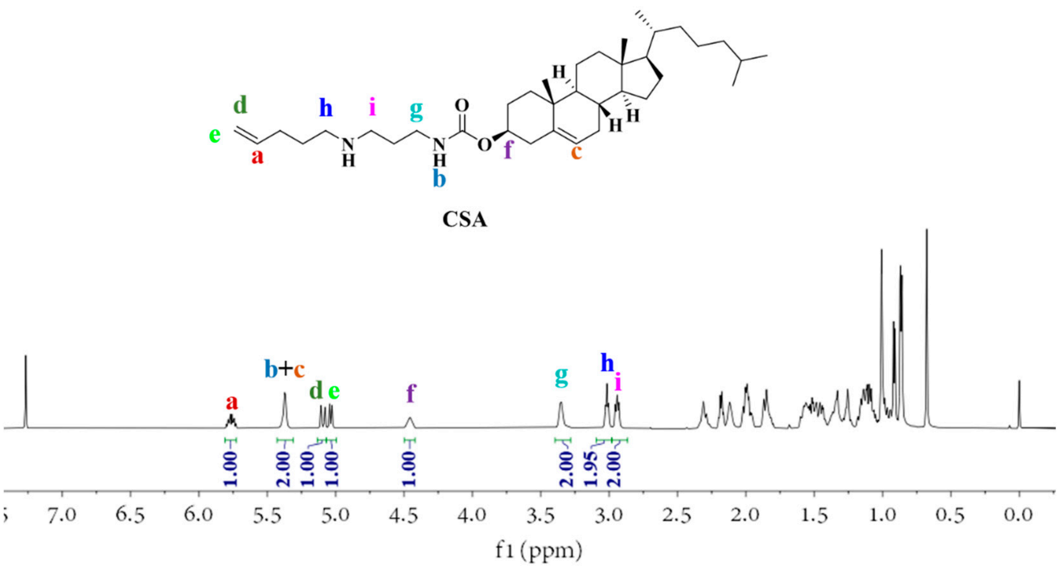

**Figure S3** $^{1}$H NMR spectrum of CSA.

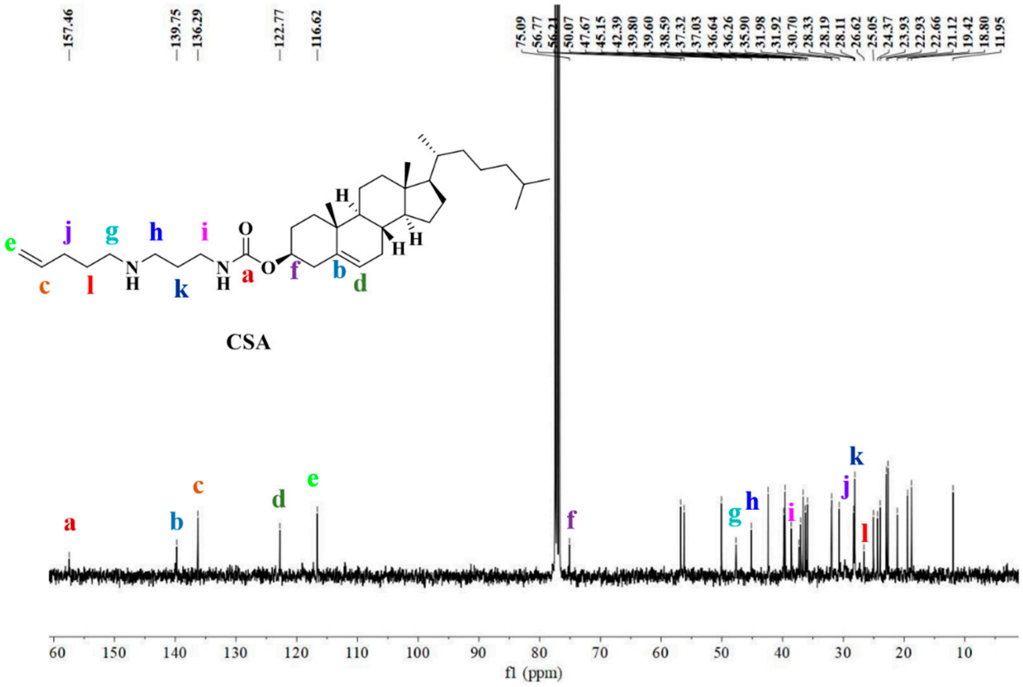

**Figure S4** $^{13}$C NMR spectrum of CSA.

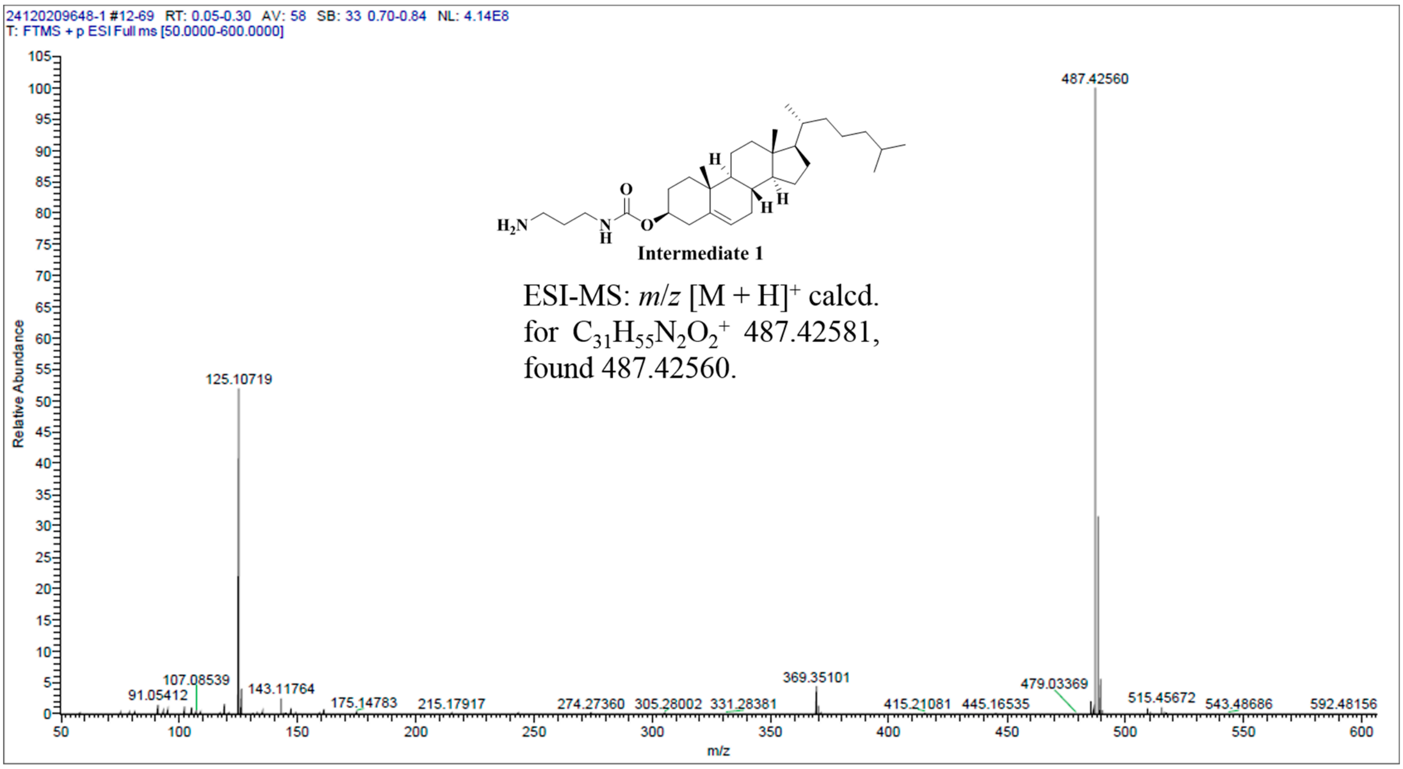


**Figure S5** ESI mass spectrum of intermediate 1.

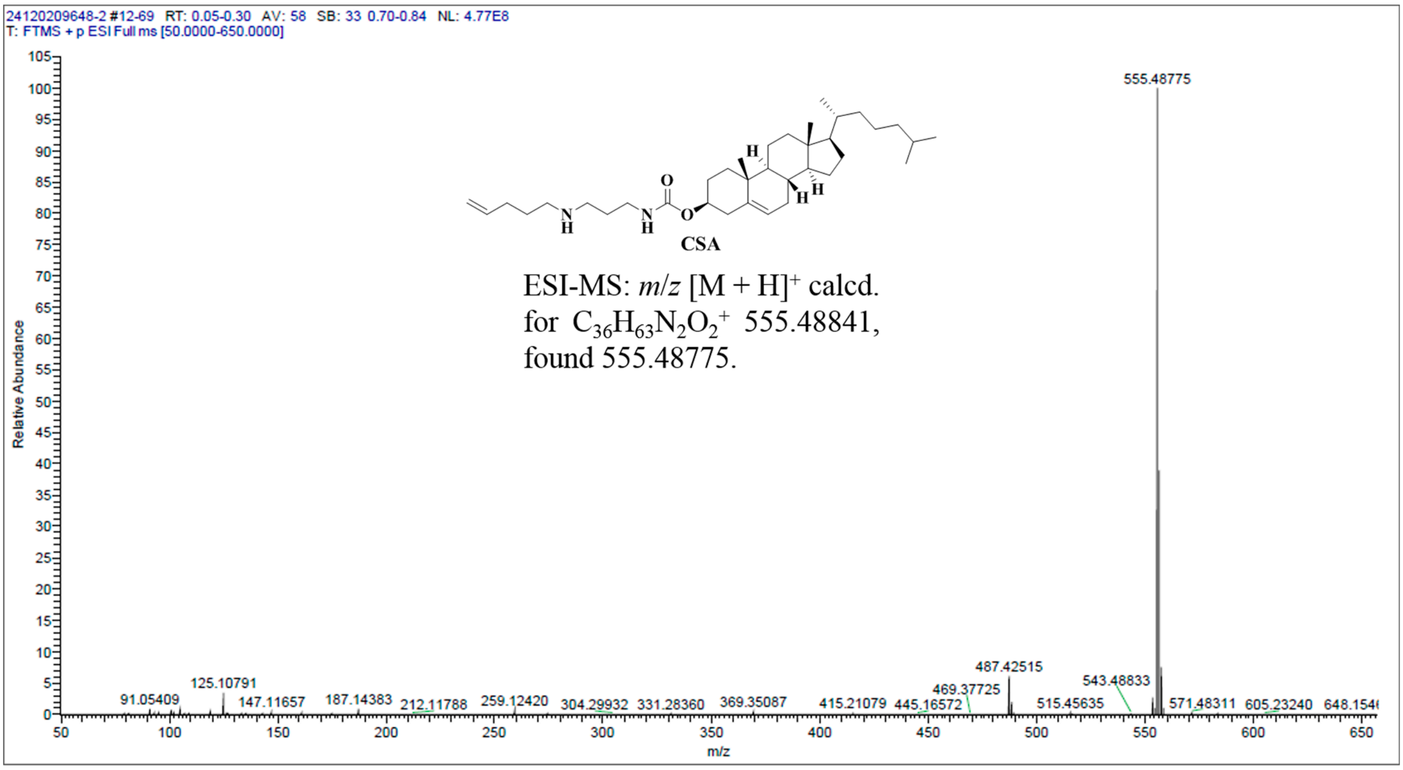


**Figure S6** ESI mass spectrum of CSA.

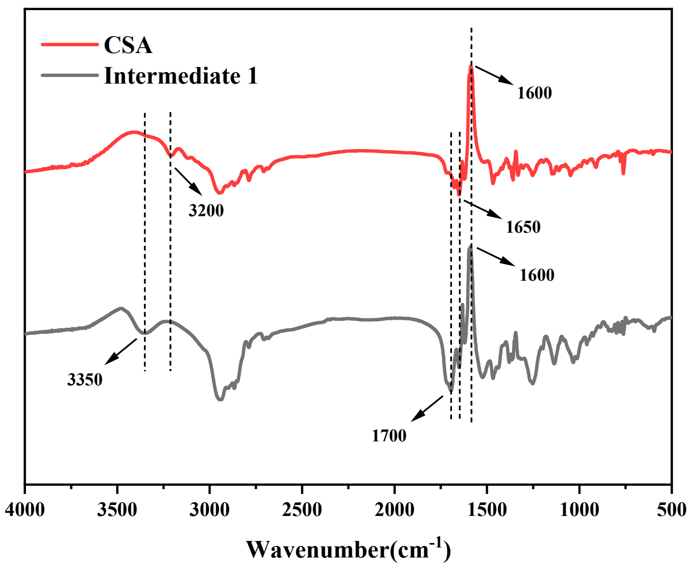


**Figure S7** The FTIR spectra of intermediate 1 and CSA.

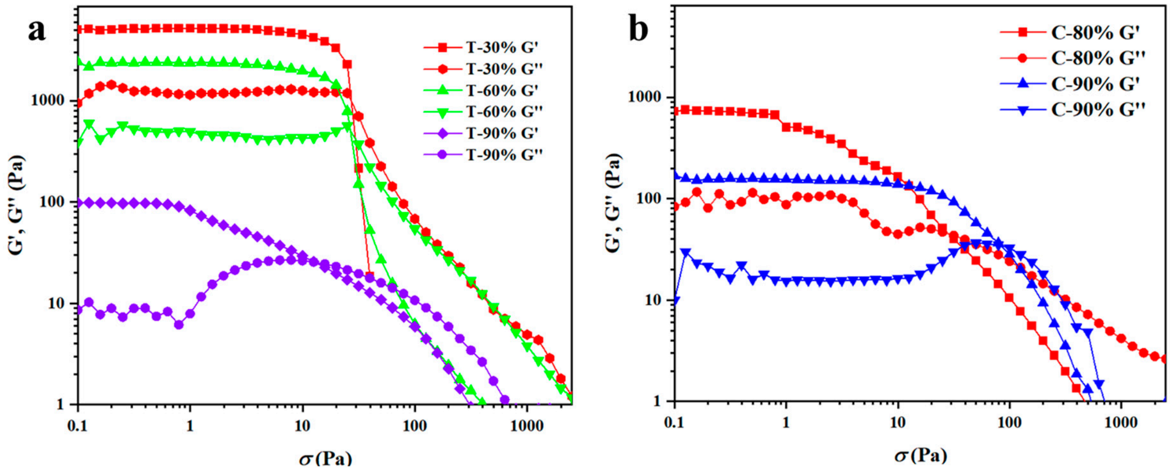


**Figure S8** (a) The variation curves of G' and G'' with shear stress for CSA/toluene/water gel emulsions with different water contents; (b) The variation curves of G' and G'' with shear stress for CSA/cyclohexane/water gel emulsions with different water contents.

## 3. References


1 Xue, M.; Miao, Q.; Fang, Y. Synthesis and gelation properties of cholesterol-based new low-molecular-mass gelators. *Acta Phys*. *Chim*. *Sin*. 2013, *29*, 2005-2012.

2 Chen, Z.; Yuan W. *N*-cyanation of primary and secondary amines with cyanobenzio-doxolone (CBX) reagent. *Chem*. - *Eur*. *J*. 2021, *27*, 14836-14840.
